# Supplementary material for: The phased pan-genome of tetraploid European potato
Source: Nature. 2025 Apr 16;642(8067):389–97. doi: 10.1038/s41586-025-08843-0 (PMC12158759; doi:10.1038/s41586-025-08843-0)
Supplement: Supplementary file 5 — Supplementary Figs. 3–14, 20, 21, 23–27 and 34–44. [file 41586_2025_8843_MOESM5_ESM.zip › suppl_figure_21/suppl_figure_21g_chr07.pdf]

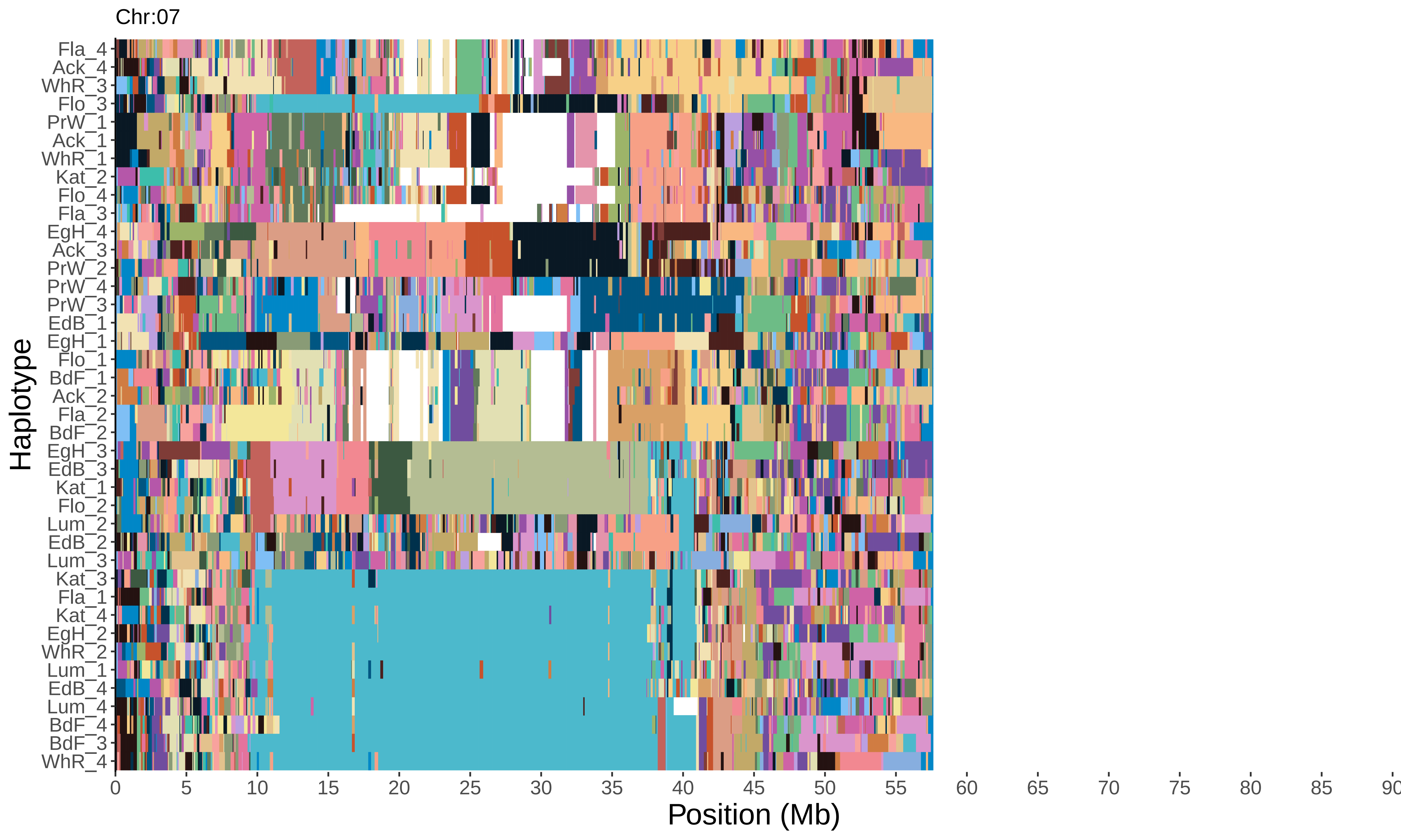
Chr:07
Haplotype
Fla_4
Ack_4
WhR_3
Flo_3
PrW_1
Ack_1
WhR_1
Kat_2
Flo_4
Fla_3
EgH_4
Ack_3
PrW_2
PrW_4
PrW_3
EdB_1
EgH_1
Flo_1
BdF_1
Ack_2
Fla_2
BdF_2
EgH_3
EdB_3
Kat_1
Flo_2
Lum_2
EdB_2
Lum_3
Kat_3
Fla_1
Kat_4
EgH_2
WhR_2
Lum_1
EdB_4
Lum_4
BdF_4
BdF_3
WhR_4
0
5
10
15
20
25
30
35
40
45
50
55
60
65
70
75
80
85
90
Position (Mb)
